# IgG Induced by Vaccination With *Ascaris suum* Extracts Is Protective Against Infection

**DOI:** 10.3389/fimmu.2018.02535

**Published:** 2018-11-09

**Authors:** Ana Clara Gazzinelli-Guimarães, Pedro Henrique Gazzinelli-Guimarães, Denise Silva Nogueira, Fabrício Marcus Silva Oliveira, Fernando Sérgio Barbosa, Chiara Cássia Oliveira Amorim, Mariana Santos Cardoso, Lucas Kraemer, Marcelo Vidigal Caliari, Milena Apetito Akamatsu, Paulo Lee Ho, Kathryn Marie Jones, Jill Weatherhead, Maria Elena Bottazzi, Peter J. Hotez, Bin Zhan, Daniella Castanheira Bartholomeu, Remo Castro Russo, Lilian Lacerda Bueno, Ricardo Toshio Fujiwara

**Affiliations:** ^1^Instituto de Ciências Biológicas, Universidade Federal de Minas Gerais, Belo Horizonte, Brazil; ^2^BioIndustrial Division, Butantan Institute, Sao Paulo Secretary of Health, São Paulo, Brazil; ^3^Texas Children's Hospital Center for Vaccine Development, Department of Pediatric Tropical Medicine, National School of Tropical Medicine, Baylor College of Medicine, Houston, TX, United States

**Keywords:** *Ascaris*, vaccine, antigens, immune response, humoral response

## Abstract

Human ascariasis has a global and cosmopolitan distribution, and has been characterized as the most prevalent neglected tropical disease worldwide. The development of a preventive vaccine is highly desirable to complement current measures required for this parasitic infection control and to reduce chronic childhood morbidities. In the present study, we describe the mechanism of protection elicited by a preventive vaccine against ascariasis. Vaccine efficacy was evaluated after immunization with three different *Ascaris suum* antigen extracts formulated with monophosphoryl lipid A (MPLA) as an adjuvant: crude extract of adult worm (ExAD); crude extract of adult worm cuticle (CUT); and crude extract of infective larvae (L3) (ExL3). Immunogenicity elicited by immunization was assessed by measuring antibody responses, cytokine production, and influx of tissue inflammatory cells. Vaccine efficacy was evaluated by measuring the reductions in the numbers of larvae in the lungs of immunized BALB/c mice that were challenged with *A. suum* eggs. Moreover, lung physiology and functionality were tested by spirometry to determine clinical efficacy. Finally, the role of host antibody mediated protection was determined by passive transfer of serum from immunized mice. Significant reductions in the total number of migrating larvae were observed in mice immunized with ExL3 61% (*p* < 0.001), CUT 59% (*p* < 0.001), and ExAD 51% (*p* < 0.01) antigens in comparison with non-immunized mice. For the *Ascaris* antigen-specific IgG antibody levels, a significant and progressive increase was observed with each round of immunization, in association with a marked increase of IgG1 and IgG3 subclasses. Moreover, a significant increase in concentration of IL-5 and IL-10 (pre-challenge) in the blood and IL-10 in the lung tissue (post-challenge) was induced by CUT immunization. Finally, ExL3 and CUT-immunized mice showed a marked improvement in lung pathology and tissue fibrosis as well as reduced pulmonary dysfunction induced by *Ascaris* challenge, when compared to non-immunized mice. Moreover, the passive transfer of specific IgG antibodies from ExL3, CUT, and ExAD elicited a protective response in naïve mice, with significant reductions in parasite burdens in lungs of 65, 64, and 64%, respectively. Taken together, these studies indicated that IgG antibodies contribute to protective immunity.

## Introduction

Recent studies estimate that ~800 million people are infected with *Ascaris lumbricoides or Ascaris suum* worldwide, which is directly related to extreme poverty, lack of basic sanitation, and health education (1). Human ascariasis, a major STH infection, has a cosmopolitan distribution and is characterized as the most prevalent neglected tropical disease in the world, affecting rural areas of low and low-middle income countries of Latin America, Caribbean, Sub-Saharan Africa, and Southeast Asia (2). In addition to rural areas, it is common to find human ascariasis in both urban and rural environments because of the hardiness of *Ascaris* eggs (3). The major human morbidities from ascariasis include intestinal obstruction, malnutrition, growth stunting, and cognitive delays from the presence of adult worms in the gut, as well as asthma from *Ascaris* larval pulmonary migrations (4, 5).

Mass drug administration programs with benzimidazole anthelminthics are currently the main strategy to control infection in humans globally. However, *Ascaris* eggs are nearly ubiquitous in the environment in developing countries, which limits the ability of mass drug administration programs to interrupt the transmission cycle within a community. As a result, post-treatment reinfection is common thereby thwarting global control and elimination efforts (6). In addition, the persistent, repetitive treatment with benzimidazole therapy may result in complications including the development of drug resistance (7) as well as residual drug deposition in animal products and in the environment (6). This approach justifies research for an alternative way to prevent and/or control ascariasis including the development of an ascariasis vaccine or a pan-anthelminthic vaccine that simultaneously targets multiple soil-transmitted helminth species (8).

Over the last decade, some studies on crude extracts (9–13) or recombinant proteins and molecules (14–17) have been addressed to identify potential candidates for an *Ascaris* vaccine. However, despite the promising performance in reducing the parasite burden in mice after immunization, the mechanisms involved in protection are still poorly understood. In this context, the objective of the present study was to determine whether vaccination with immunogenic *Ascaris* crude antigens mediated protection after *Ascaris* challenge. Our results revealed that the immunization induced by crude extracts elicits host protection against Ascaris infection with preserved lung physiology through a humoral-dependent response, which is associated with production of IL-5 and IL-10.

## Materials and methods

### Parasites

*Ascaris suum* adult worms werHe collected from intestines of infected pigs that were discarded by a slaughterhouse located in the city of Belo Horizonte, Minas Gerais, Brazil. Adult worms were kept in PBS (0.4 M NaCl and 10 mM NaPO4) and taken to the Laboratory of Immunology and Genomics of Parasites of the Federal University of Minas Gerais to be processed. The eggs were isolated from the uteri of female adult worms by mechanical maceration, purified by filtration on 100 μm nylon strainers, placed in culture bottles with 50 mL of 0.2 M sulfuric acid at a concentration of 25 eggs/μL and maintained in BOD incubator at 26°C. At the 150th day of culture, the peak of larvae infectivity, the fully embryonated eggs were used for experimental infections (18).

### *In vitro* induction of larvae hatching for larval crude extract production

To induce the hatching of fully embryonated *A*. *suum* eggs, a modified standard protocol for *Toxocara canis* was used (19). After keeping the eggs in 0.2 M sulfuric acid for 150 days, the eggs were subsequently centrifuged at 600 g for 10 min at room temperature (RT). After the acid was discarded, the eggs were resuspended in 5% (v/v) sodium hypochlorite solution and incubated at 37°C and 5% CO_2_ for 2 h to rupture the outer egg membrane. After incubation, the fully embryonated eggs were separated from the non-embryos by centrifugation at 800 g for 10 min at room temperature (RT). The embryonated eggs were concentrated in the supernatant, washed three times in 20 mL PBS by centrifugation at 800 g for 10 min at room temperature (RT). The pellet containing the embryonated eggs was resuspended in Hank's solution pH 2.0, followed by incubation for 30 min at 37°C and 5% CO_2_. After incubation, the embryonated eggs were centrifuged at 800 g for 10 min. The egg pellet was resuspended in RPMI-1640 medium (SIGMA, USA), pH 7.2, supplemented with 4% penicillin/streptomycin (Invitrogen, USA) and placed in 24 wells plates for larvae hatch. The culture was maintained for seven days at 37°C and 5% CO_2_. Following incubation, the L3 larvae were purified.

### Production of crude extracts of adult worm, adult worm cuticle, and infective larvae

The crude extract of adult worm (ExAD) was initially obtained by mechanical maceration of the parasites in PBS and then by using a sonicator (Cole Parmer Ultrasonic Homogenizer Power Supply 4710 Series, USA). The macerated extract, kept cool on ice, was sonicated at 60 Watts for 1 min, with a 30 s interval for each cycle, totalling five cycles. Thereafter, the soluble crude extract was purified by centrifugation at 800 g for 15 min at 4°C. The pellet was then discarded and the supernatant stored at −80°C until use. Using the protocol described above for the ExAD, crude extract of the adult worm cuticle (CUT) was obtained using only the cuticle of the adult worms in the first step of the mechanical maceration of the parasite.

Finally, to produce the crude extract of L3 larvae (ExL3), purified larvae (section *In vitro* Induction of Larvae Hatching for Larval Crude Extract Production) were collected and transferred to a 50 mL graduated tube where they were centrifuged at 800 g for 10 min at room temperature (RT). The supernatant was discarded and the pellet was resuspended in 5 mL of PBS, and then sonicated at 60 Watts for 1 min, with a 30 s interval each cycle, for a total of 10 cycles. After sonication, the extract was centrifuged at 800 g for 15 min at 4°C. The supernatant was collected and stored at −80°C until use. The amount of protein in all antigenic extracts was measured using commercial BCA kit (Thermofisher Scientific, USA), performed according to the manufacturer's instructions.

### Antigen characterization

To characterize the antigens, the samples were subjected to separation by electrophoresis in polyacrylamide gel using 40% bis-acrylamide. The 12.5% separation gel was prepared using 1.5 M Tris-HCl pH 8.8 and 0.4% SDS; 0.5% (v/v) ammonium persulfate and 0.05% (v/v) TEMED. The concentration gel was prepared similarly to the separation but using the 0.5 M Tris HCl buffer pH 6.8. It was applied to the 10 μg channels of each antigen along with the molecular weight standard. Electrophoresis was performed in 25 mM Tris HCl run buffer; 192 mM glycine; 0.1% SDS and pH 8.3; to the constant voltage of 100 Volts. After the run was completed, the gels were stained and incubated for 2–16 h with Coomassie Blue solution (Coomassie Brilhant Blue G-250 0.1%, methanol 50%, acetic acid 10%), and then bleached in solution containing 30% methanol and 10% acetic acid. The photo documentation was performed with the assistance of ImageQuant LAS 4000 equipment (GE Healthcare Life Science, USA).

### Active vaccination protocol and *ascaris* infection

BALB/c mice (male, 7 weeks-old) were obtained from the Central Animal Facility—Federal University of Minas Gerais, Brazil. The experimental design was as follows (Supplementary Figure 1): mice were divided into six groups containing 15 mice per group. The first group (G1) was composed of non-immunized and non-infected control mice (PBS NI); the second group (G2) was composed of mice immunized with PBS alone; the third group (G3) was immunized with *Bp*MPLA adjuvant (Monophosphoryl lipid A from *Bordetella pertussis*, Butantan Institute, Brazil); the fourth group (G4) was immunized with *Ascaris suum* larval crude extract antigen (ExL3) added to the MPLA adjuvant; the fifth group (G5) was immunized with crude adult worm extract antigen (ExAD) combined with MPLA adjuvant; and the sixth group (G6) was immunized with crude cuticle extract (CUT) with the MPLA adjuvant. For each immunization, 25 μg of each antigen was administered along with 25 μg of MPLA adjuvant subcutaneously in a total volume of 300 μL per animal. In total, mice were submitted to three immunizations at 10 days intervals, followed by challenge with infective *Ascaris* eggs by oral gavage 10 days after the final immunization.

For the *Ascaris* infection, prior to inoculation, the fully embryonated eggs were incubated with 5% (v/v) sodium hypochlorite solution in an incubator (37°C and 5% CO_2_) for 2 h to disrupt the outer layer of the eggs and, therefore, to facilitate *in vivo* larval hatching. After the incubation, the eggs were resuspended and washed with PBS 5 times. The mice of the groups G2, G3, G4, G5, and G6 were inoculated by oral gavage with 0.2 mL of the solution containing 2,500 embryonated eggs_._

### ELISA for detection of specific antibody production

ELISA assays were performed using mouse sera after three sequential immunizations with ExAD, CUT, and ExL3 and control (MPLA). The sera were collected before the first immunization, 10 days after the first immunization, 10 days after the second immunization and 10 days after the third immunization. To measure specific IgG and IgG subclasses, ELISA plates (Greiner-Bio-One, USA) were coated with 1 μg/well of ExAD antigen diluted in carbonate buffer and left overnight at 4°C. The following day, plates were washed 8 times with washing buffer (PBS-0.05% Tween20) and blocked with 250 μL of PBS/BSA 3% for 1 h at 37°C. After blocking, the entire well solution was removed by aspiration. Subsequently, 100 μL of 1:1,000 diluted sera in PBS/BSA 3% were added to the wells and incubated at 4°C overnight. The next day, plates were washed 8 times with washing buffer and 100 μL of peroxidase conjugated anti-mouse IgG antibody (Sigma-Aldrich, USA) diluted 1: 2,000 in PBS/BSA 3% was added. After 1 h incubation at 37°C, the plates were washed again, and 100 μL of the developing solution containing 0.1 M citric acid, 0.2 M Na_2_PO, 0.05% OPD (o-phenylenediamine dihydrochloride) and 0.1% H_2_O_2_ was added. The plates were incubated at 37°C for 20 min and the reaction was stopped by the addition of 50 μL 0.2 M sulfuric acid. The resulting absorbance was read on ELISA reader (VersaMax ELISA Microplate Reader/Molecular Devices, USA) using Softmax Pro 5.3 software at 492 nm. All assays were performed in duplicates. To measure *Ascaris*-specific IgE and IgA levels, the protocol described above was used, however, the sera dilution was 1:10 and 1:20, respectively.

*Ascaris summ* recombinant proteins As14 and As16, previously demonstrated in the literature as promising vaccine candidates (15), were provided by Dr. Bin Zhan from the Texas Children's Hospital Center for Vaccine Development, Baylor College of Medicine, Houston, TX, USA, to test reactive antibodies from mice immunized with ExL3, ExAD, and CUT *Ascaris* antigen would recognize the targets as major antigens. ELISA plates (Greiner-Bio-One, USA) were coated with 3 μg/well of As14 and As16 proteins and left overnight at 4°C. The ELISA protocol described above was utilized to quantitate specific IgG antibody to As14 and As16 proteins.

### Parasitological analysis

Parasite burdens from immunized and non-immunized mice were evaluated by counting the total number of larvae recovered in the lungs at day 8 post-infection (*n* = 15 mice/group). On day 8 post-infection, the mice were euthanized and the lungs were collected, punctured with surgical scissors and placed in a modified Baermann apparatus for 4 h in PBS at 37°C and 5% CO_2_. The larvae recovered in the pellet of the apparatus were fixed in 4% formalin and quantified by light microscopy.

### Bronchoalveolar lavage (BAL)

Mice were anesthetized and a 1.7 mm catheter was inserted into the trachea. One mililiter of PBS was flushed twice through the catheter to collect bronchoalveolar lavage fluid. The lavage fluid was filtered to purify *A. suum* larvae presented in the BAL, centrifuged at 3,000 g for 10 min and the supernatants were collected; the pellet was used to quantitate the total and differential cellularity using light microscopy.

### Cytokine profiles

To determine the serum cytokine profiles, 500 μL of blood were collected from each animal at every experimental timepoint: (1) before first immunization; (2) after the first immunization; (3) after the second immunization; and (4) after the third immunization. Blood was collected from the retro-orbital sinus using a Pasteur capillary pipette without anticoagulant and placed in coagulation microtubes, followed by centrifugation and serum collection. The production of IL-2, IL-5, IL-6, IL-10, IL-13, and TNF-α was measured using the Luminex Milliplex Th1/Th2/Th17 kit according to the manufacturer's determination (Millipore, USA).

To determine the tissue cytokine profile, the right lobe of the lung was removed from 7 mice from each group and 100 mg of tissue was homogenized by the Power Gen 125 tissue homogenizer (Fisher Scientific, USA) in 1 mL of PBS supplemented with protease inhibitors (0.1 mM phenyl methyl sulfonyl fluoride, 0.1 mM benzethonium chloride, 10 mM EDTA, and 20 KI aprotinin A) and 0.05% Tween20. The homogenate was centrifuged at 800 g for 10 min at 4°C and the supernatant was used to determine the cytokines IL-5, IFN-γ, IL-10, TGF-ß by ELISA (R&D Systems, USA) according to the manufacturer's protocol.

### Assessment of respiratory mechanics

Mice were anesthetized with a subcutaneous injection of ketamine and xylazine (8.5 mg/kg xylazine and 130 mg/kg ketamine) to maintain spontaneous breathing under anesthesia. Mice were tracheostomized, placed in a plethysmograph and connected to a computer-controlled ventilator (Forced Pulmonary Maneuver System®, Buxco Research Systems, Wilmington, North Carolina USA). The mice were ventilated at a rate of 300 breaths per minute. After 3 min of ventilation, the constant-phase model was used to measure Total Lung Capacity (TLC), Functional Residual Capacity (FRC), Residual Volume (RV), Dynamic Compliance (Cdyn), and Lung Resistance (Rl), as previously described (20). At the end of the experiment, the mice were euthanized and the organs were collected for further analysis.

### Histopathological analysis

The quantification of inflammatory cells in the lung tissue was performed by morphometric analysis of the left lobe of the lung after staining with hematoxylin and based on images from 40 randomly selected fields (total area: 1.12 × 10^6^ μm^2^) of the histological sections per animal. The inflammatory infiltrate in the lung was quantified by counting the number of nuclei present in the histological sections. Histological changes were evaluated in the lungs of antigen-immunized and control mice post-*Ascaris* challenge, during the peak of larvae migration. The left lobe of the lungs was removed from the mice in each group. The organs were fixed in 4% formalin solution, gradually dehydrated in ethanol before being diaphanized in xylol, and included in paraffin blocks that were cut at 4–5 microns thick and fixed on the microscopy slide. Slides with lung tissue were stained with hematoxylin and eosin for evaluation of tissue damage, and with Gomori stain for assessment of tissue deposition of collagen. The lesions exhibited in the pulmonary parenchyma were described in terms of lesion intensity, inflammation, and vascular phenomena.

For semiquantitative analysis, the slides were examined under a light field optical microscope coupled to a digital image capture system (Motic 2.0). To determine the airway inflammation score, using perivascular inflammation, parenchymal inflammation, and hemorrhage, 10 random images per animal were captured and analyzed (10x). The score was made from adaptations of the methodology previously described by Horvat et al. (21) (Supplementary Table 1).

### Evaluation of antibody-mediated protection by passive transfer of purified immunoglobulins from sera of immunized mice

To obtain purified immunoglobulins, a pool of 3 mL of sera from mice immunized with each of the three extracts was made. Sera samples were subjected to protein A conjugated affinity chromatography which binds the Fc portion of the IgG immunoglobulin. Chromatography was based on the binding of antibodies to protein A column at pH 8.6. After ligation, the column was washed using a 50 mM Tris-HCl; 0.5 M NaCl solution. The eluted fractions containing the purified immunoglobulin were recovered and quantified by the commercial BCA kit (Thermofisher Scientific, USA), performed according to the manufacturer's instructions. After the immunoglobulin was quantified, the passive transfer experiment was conducted in *n* = 6 male BALB/c mice (8 weeks of age) per group, through three intraperitoneal injections totalling 52 μg of IgG immunoglobulin transferred into the respective groups. Mice in the control group received only MPLA adjuvant. Before euthanasia, the serum of these mice were collected for further analysis. The experimental design is shown in Figure 8A.

### Statistical analysis

GraphPad Prism 6 (Graphpad software, Inc., USA) was used for the statistical analysis. The Grubb's test was used to detect sample outliers (in parametric analysis), and non-parametric analysis of all the results was performed. For the comparisons between the parasite burdens, IgG immunoglobulin production, lung lesion areas, the Kruskal-Wallis test followed by Dunn's test was used. For the comparison of the production of systemic and tissue cytokines, the Kruskal-Wallis test followed by Dunn's test and Tukey's Multiple Comparison Test were used. Newman-Keuls multiple comparison test was used for comparison of pulmonary mechanics. All tests were considered significant when *p* ≤ 0.05.

### Ethics statement

The maintenance and use of mice was carried out in accordance with the recommendations of the Brazilian College of Animal Experimentation (COBEA). The present study was submitted and approved by the Ethics Committee for Animal Experimentation (CEUA) of the Federal University of Minas Gerais, Brazil through protocol # 187/2014. All efforts were made to minimize animal suffering.

## Results

### Immunization with *ascaris suum* antigens induced antibody responses in mice

After the ExL3, ExAD, and CUT had been produced, their antigen profile was assessed by protein gel electrophoresis (Supplementary Figure 2), prior to actively immunizing mice with each of the three antigens extract, respectively, together with the adjuvant MPLA.

**Figure 1:**
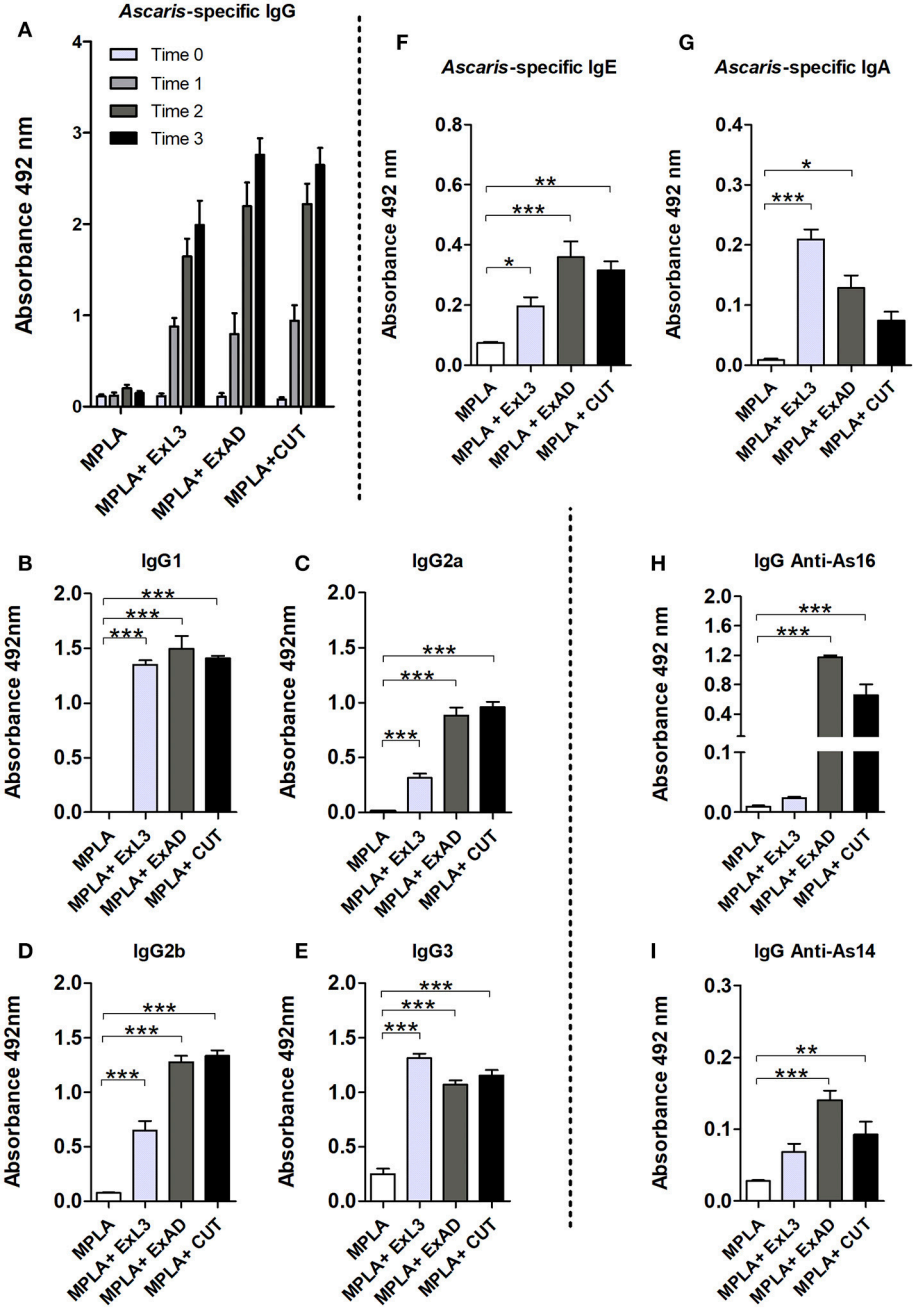
Experimental immunization with *Ascaris suum* antigen-induced antibody responses. Antigen-specific antibodies from immunization with extracts of the infective larvae L3 (ExL3), adult worms (ExAD), and the cuticle of adult worms (CUT) were measured by ELISA in BALB/c mice during and after immunization. **(A)** *Ascaris***-**specific IgG before first immunization (Time 0), post first immunization (Time 1), post second immunization (Time 2), and post three immunizations (Time 3). IgG1 **(B)**, IgG2a **(C)**, IgG2b **(D)**, and IgG3 **(E)** in the serum of mice after the third immunization (Time 3) with the *A. suum* antigens. **(F)** *Ascaris***-**specific IgE. **(G)** *Ascaris*-specific IgA. Specific recognition of the proteins As16 **(H)** and As14 **(I)** by antibodies of mice immunized with larval antigen (ExL3), crude extract of adult worm (ExAD), and cuticle (CUT). Statistically significant differences (*p* < 0.05) were represented by ^*^ compared to the control group MPLA in which ^*^p < 0.05, ^**^p < 0.01, ^***^p < 0.001.

In order to evaluate the antibody response against the different *A. suum* antigens, we measured the antigen-specific IgG levels in the mice. Based on the results, we observed increasing production of antigen-specific IgG depending of the number of immunizations in the protocol (Figure 1A): time 0 referring to sera collected in the time before the first immunization; time 1, after the first immunization; time 2, after the second immunization, and time 3, after the third immunization. All mice immunized with an *Ascaris*-specific antigen showed a significant increase in antigen-specific IgG production when compared to the control adjuvant MPLA group.

After observing a significant increase in antigen-specific IgG and an approximate 60% parasite burden reduction in the antigen-immunized group, we investigated the contribution of IgG subclasses in the sera of the mice within each group. We observed high levels of IgG1, IgG2a, IgG2b, and IgG3 in the serum of immunized mice. IgG1 (Figure 1B) and IgG3 (Figure 1E) showed a significant increase in all immunized groups compared to the control group. IgG2a and IgG2b subclasses were also detected at high levels in the serum of the immunized mice. However, these subclasses were found predominating in the groups immunized with crude adult worm extract (ExAD) and *A. suum* cuticle (CUT), but at low levels in the serum of the mice immunized with larval antigens (ExL3) (Figures 1C,D). In order to evaluate the production of other antibody isotypes in the sera of the mice after immunization, *Ascaris*-specific IgE and IgA levels were also measured. A significant increase in the production of antigen-specific IgE was observed in all immunized groups when compared to the control group (Figure 1F). Likewise, a significant increase in the production of antigen-specific IgA (Figure 1G) was measured in mice immunized with ExL3 and ExAD, with a higher production of IgA in the ExL3 group. No statistical difference was observed in the IgA production in the CUT-immunized group compared to the control.

After the characterization of antigen-specific antibody levels, the reactive antibodies from ExL3-, ExAD-, and CUT-immunized mice were evaluated to determine if these antibodies would recognize the recombinant proteins As16 and As14 as major antigens. IgG antibodies from mice immunized with ExAD and CUT showed strong recognition of As16 in comparison to antibodies from ExL3 immunized mice or controls (*p* < 0.01 and *p* < 0.001, respectively) (Figure 1H). In contrast, As14 was much less reactive (Figure 1I).

### Immunization with different *A. suum* extracts reduces the parasite burden in BALB/c infected mice

**Figure 2:**
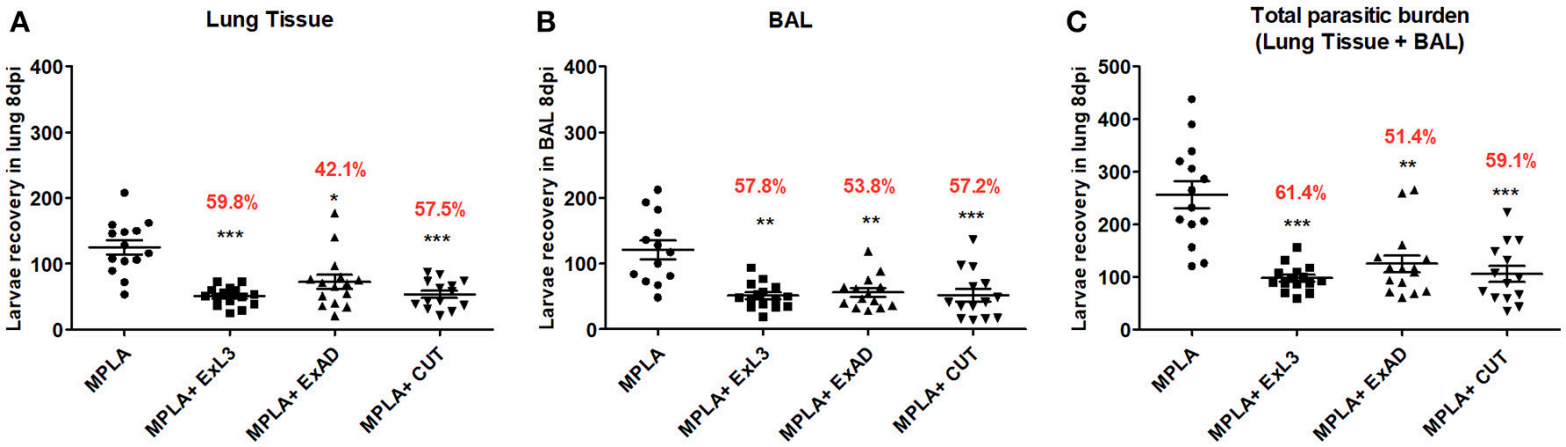
Immunization with different *A. suum* extracts reduces the parasite burden in infected BALB/c mice. The parasite burden was quantified by a modified Baermann apparatus after 8 days of infection in BALB/c mice. **(A)** Number of larvae recovered in the lung tissue; **(B)** Number of larvae recovered in the bronchoalveolar lavage; **(C)** Total number of parasite burden (Lung tissue+BAL). Statistically significant differences (*p* < 0.05) were plotted compared to the MPLA control group in which ^*^p < 0.05, ^**^p < 0.01, ^***^p < 0.001. The percentage value shown in the graph indicates the mean reduction of the parasitic burden in relation to the control group (MPLA).

In order to evaluate the clinical efficacy of *Ascaris*-specific antigen immunization, we initially measured the number of larvae recovered in the lungs and bronchoalveolar lavage (BAL) at day 8 post-*Ascaris* challenge, which corresponds with the peak of larval migration in this organ. Mice immunized with ExAD, CUT, and ExL3 combined with MPLA adjuvant showed a significant reduction in parasite burden in both the lung tissue and BAL fluid when compared to controls (Figure 2).

The larvae recovery in the lung tissue (Figure 2A) revealed that the group immunized with ExL3 showed the greatest reduction in parasitic burden when compared to control mice, reaching 59.8% (*p* < 0.001) of protection, followed by 57.5% (*p* < 0.001) of protection for mice immunized with CUT, and 42.1% (*p* < 0.05) of protection for mice immunized with ExAD.

Similarly, the larvae recovery in the BAL fluid (Figure 2B) showed a significant reduction in larvae in mice immunized with ExL3 reaching 57.8% reduction in relation to the controls (*p* < 0.01), followed by 57.2% reduction (*p* < 0.001) for the group immunized with CUT, and 53.8% reduction (*p* < 0.01) for the group immunized with ExAD.

The overall parasitic burden in each animal, at day 8 of infection, was calculated by combinding the number of larvae in the BAL fluid and in the lung tissue. The antigen ExL3 presented a total larvae reduction of 61.4% (*p* < 0.001), followed by 59.1% (*p* < 0.001) for mice immunized with CUT, and 51.4% (*p* < 0.01) for mice immunized with ExAD in comparison to control (Figure 2C).

### Cytokine levels induced by immunization with different *A. suum* extracts and following *A. suum* infection

**Figure 3:**
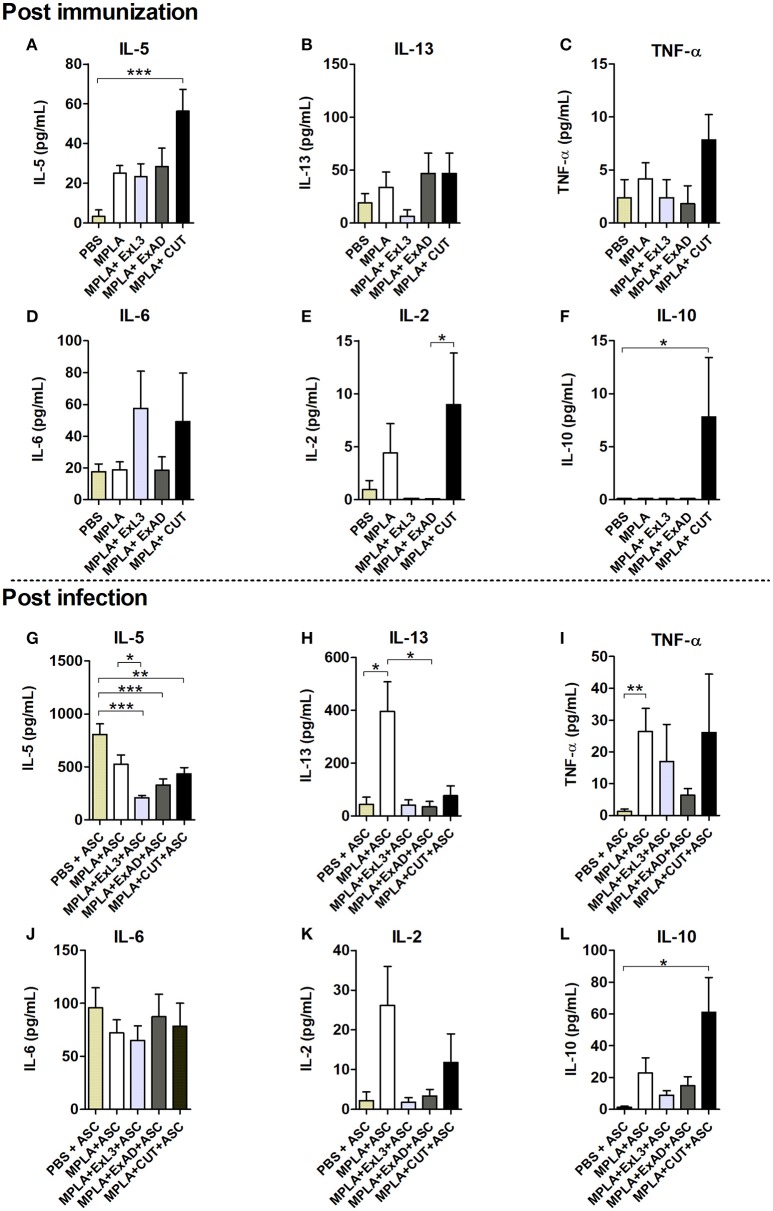
Systemic cytokine levels induced by immunization with different *A. suum* extracts following infection. Upper panel Post immunization—Determination of systemic cytokine levels by ELISA after immunization with antigens. **(A)** IL-5. **(B)** IL-13. **(C)** TNF-α. **(D)** IL-6. **(E)** IL-2. **(F)** IL-10. Bottom panel Post infection—Determination of systemic cytokine levels after immunization with antigens and infection with *A. suum*. **(G)** IL-5. **(H)** IL-13. **(I)** TNF-α. **(J)** IL-6. **(K)** IL-2. **(L)** IL-10. Statistically significant differences (*p* < 0.05) were plotted compared to the controls groups PBS and PBS-ASC in which ^*^p < 0.05, ^**^p < 0.01, ^***^p < 0.001.

After observing a marked reduction in the parasite burden in mice immunized with *A. suum* antigens, the next aim was to identify the potential mechanisms involved in this protection. Host cytokine production was characterized after the immunizations with the parasite antigens and post-infection with *A. suum*. Systemic levels of cytokines were evaluated at two timepoints: after immunization with an *A. suum* antigen (Figures 3A–F, upper panel) and after challenge infection (Figures 3G–L, bottom panel). Based on the results of the cytokine production induced by immunization, CUT antigen induced a significant increase in the IL-5 (*p* < 0.001) (Figure 3A) and IL-10 (*p* < 0.05) (Figure 3L), compared to PBS control group. There were no differences in the production of IL-13 (Figure 3B), TNF-α (Figure 3C), IL-6 (Figure 3D), and IL-2 (Figure 3F), after immunization compared to the PBS control group.

After infection we found reduced IL-5 (*p* < 0.001) levels in the immunized groups with the extracts compared to mice in the control group, which had been immunized with PBS and subsequently infected with *Ascaris* (PBS-ASC) (Figure 3G). No significant difference was observed in IL-13 (Figure 3H), TNF-α (Figure 3I), IL-6 (Figure 3J), and IL-2 levels (Figure 3K) in relation to PBS-ASC. However, there was a significant increase in systemic IL-10 production (*p* < 0.05) after *Ascaris* infection in mice immunized with CUT antigen compared to PBS-ASC controls (b).

### Immunization with different *A. suum* extracts had distinct effects on the lung inflammation following *A. suum* infection

**Figure 4:**
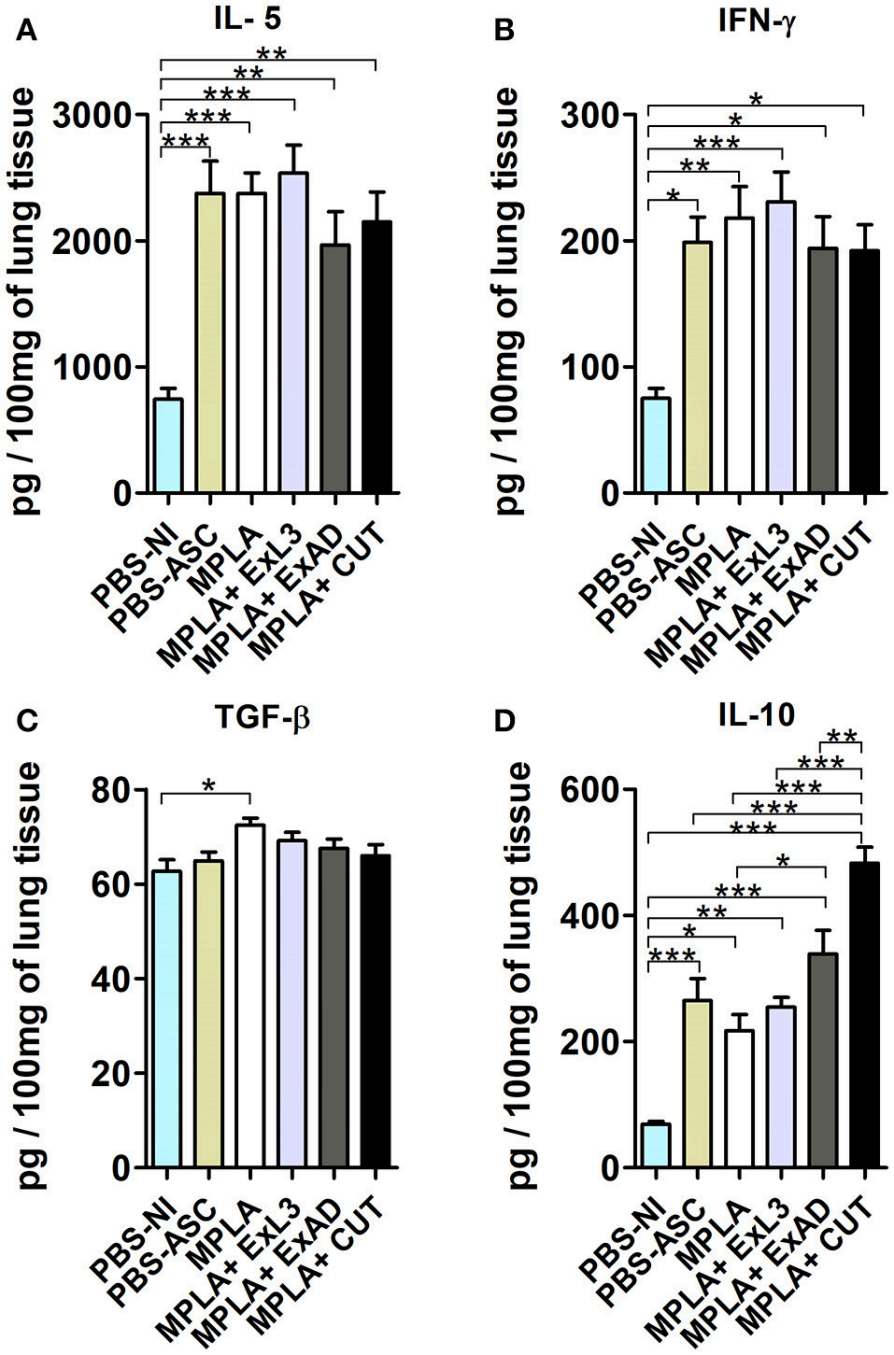
Pulmonary cytokine levels induced by immunization with different *A. suum* extracts following *A. suum* infection. The levels of cytokines were quantified by ELISA. **(A)** IL-5, **(B)** IFN-γ, **(C)** TGF-ß, and **(D)** IL-10. Statistically significant differences (*p* < 0.05) were plotted compared to the controls groups PBS-NI and PBS-ASC in which ^*^p < 0.05, ^**^p < 0.01, ^***^p < 0.001.

To characterize the immune response in the lung tissue induced by the antigen immunization, followed by *Ascaris* infection, the cytokines IL-5, IL-10, IFN-γ, and TGF-ß (Figure 4) levels were measured. *Ascaris* infection induced increased levels of IL-5, IFN-γ, and IL-10 in the lung tissue, and pre-immunization had no effect on the IL-5 and IFN-γ levels (Figures 4A–C). However, the CUT antigen-immunized and *Ascaris*-infected mice showed significant increases in IL-10 levels (*p* < 0.001) in the lungs compared to PBS-ASC controls (Figure 4D). Moreover, CUT antigen-immunized and *Ascaris*-infected mice displayed elevated lung IL-10 levels compared to MPLA, ExL3, and ExAD immunized mice (Figure 4D).

**Figure 5:**
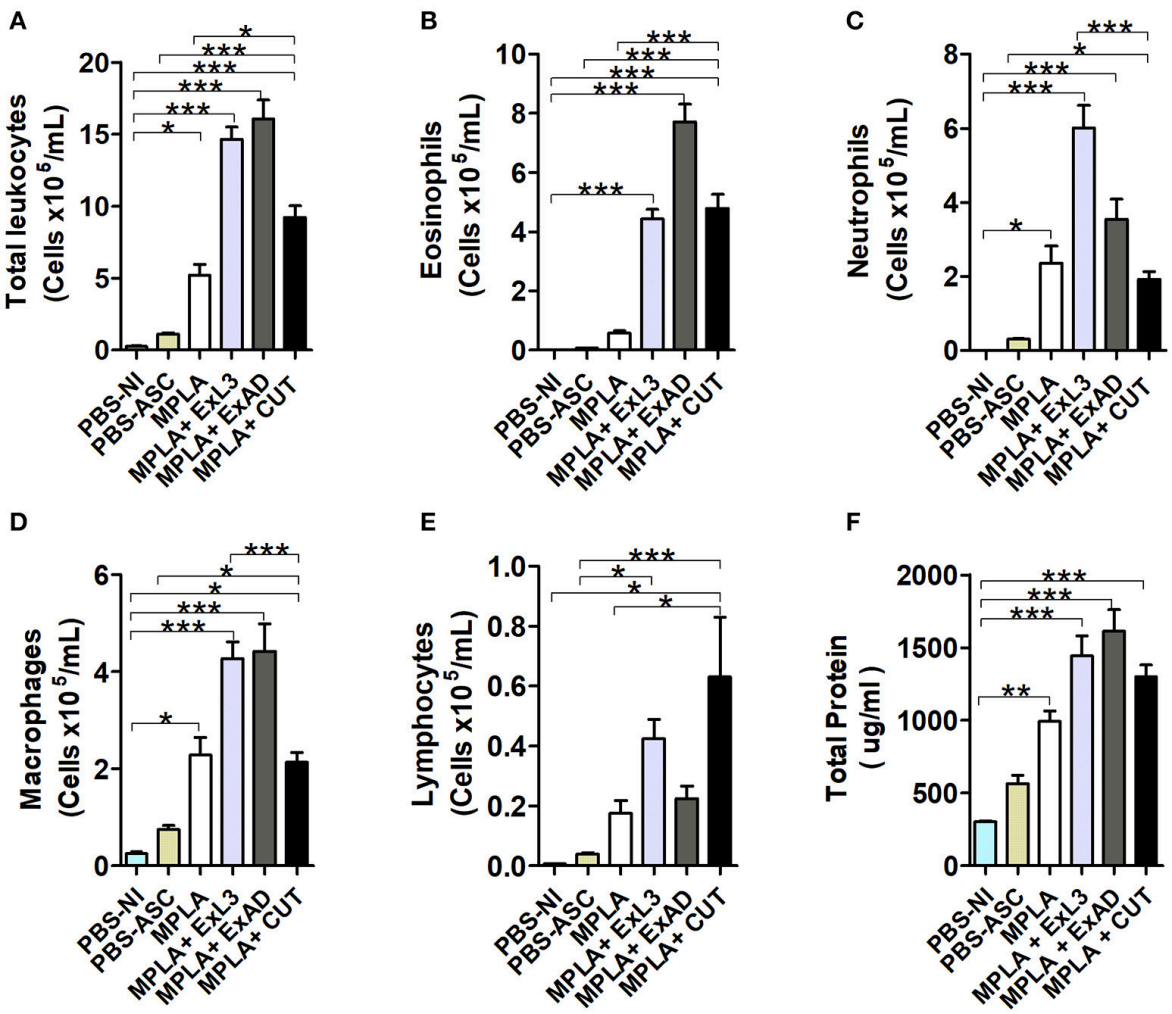
Immunization with different *A. suum* extracts had distinct effects on leukocyte influx into the airways following *A. suum* infection. Inflammatory cells in the bronchoalveolar lavage (BAL) after immunization with the extracts and challenge with *A. suum* were quantified using a Neubauer chamber and cytospin preparations. **(A)** Total leukocytes. **(B)** Eosinophils. **(C)** Neutrophils. **(D)** Macrophages. **(E)** Lymphocytes. **(F)** Total protein. Statistically significant differences (*p* < 0.05) were plotted compared to the control groups PBS-NI and PBS-ASC in which ^*^p < 0.05, ^**^p < 0.01, ^***^p < 0.001.

After characterizing the tissue cytokine profile driven by antigen immunization, BAL was evaluated to characterize leukocyte influx into the airways in response to *A. suum* infection (Figure 5). An increase in the total number of leukocytes was found in the MPLA-immunized group, but highly expressed in immunized groups ExL3, ExAD, and CUT compared to PBS-ASC (Figure 5A) challenged mice. Indeed, this increased number of leukocytes in ExL3 and ExAD immunized mice was marked by influx of eosinophils, neutrophils, macrophages, and lymphocytes into airways compared to MPLA immunized mice (Figures 5B–E). However, the CUT-immunized mice showed reduced in total leukocytes compared to ExL3 and ExAD (Figure 5A), with impact in neutrophils and macrophages in the airways (Figures 5C,D) and increased lymphocytes (Figure 5E). Protein leakage is a marker of tissue inflammation. After *A. suum* infection, we found a significant increase in protein in the airways in all groups, as measured by total protein quantification in BAL (Figure 5F).

**Figure 6:**
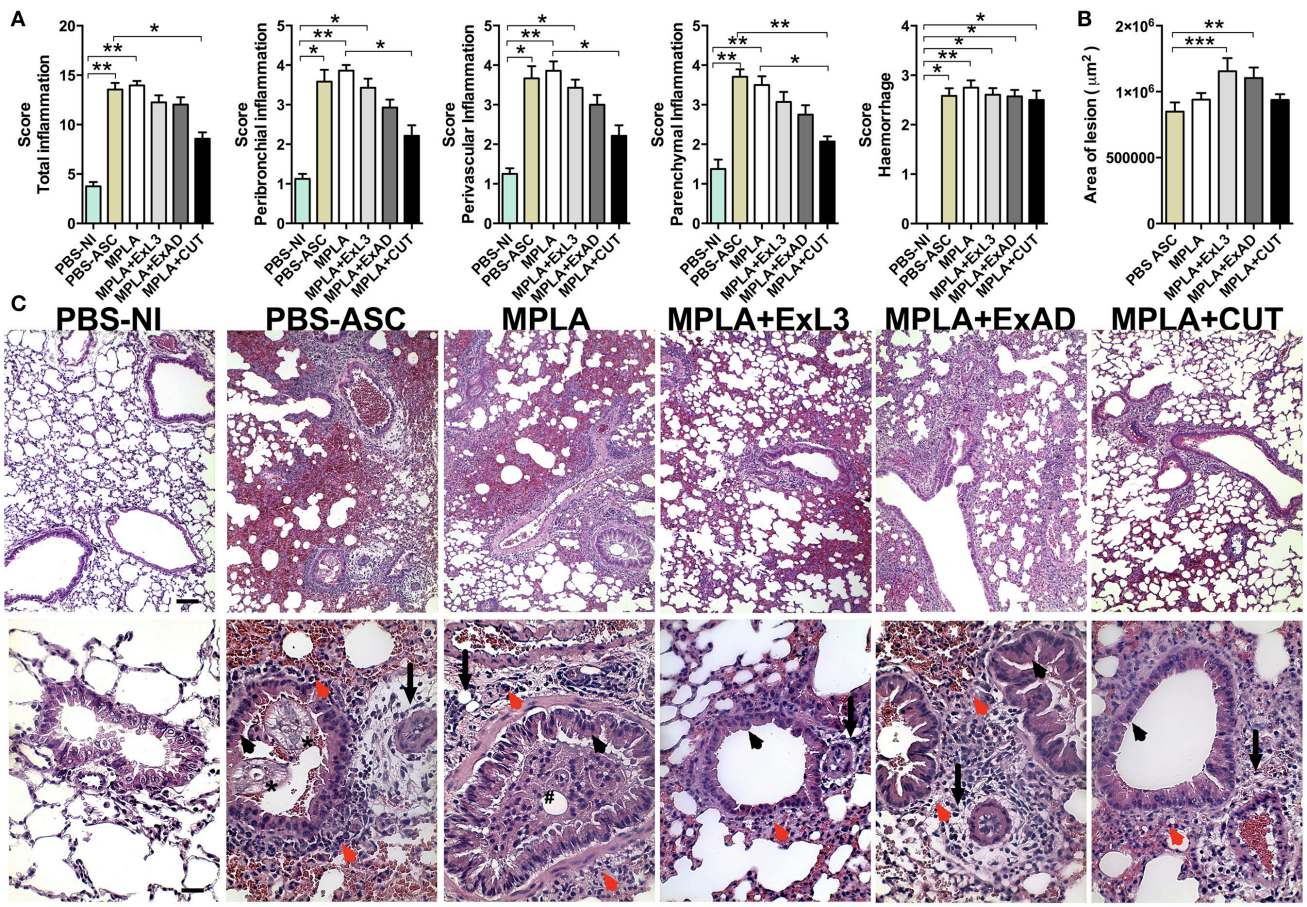
Immunization with different *A. suum* extracts had distinct effects on pulmonary inflammation following *A. suum* infection. Determination of pulmonary injury and inflammation by analysis of pulmonary **(A)** Score of inflammation; **(B)** Morphometry of total area of inflammation; and **(C)** Histopathological visualization of the lesion caused by larval migration in the lungs, perivascular edema (arrow), peribronchial cell infiltrate (red arrowhead), larvae occupying the lumen of the bronchi and bronchioles (asterisk), hypertrophy and hyperplasia of bronchial epithelial (arrowhead), mucous plug obstructing the bronchus (hashtag). Stained by H&E, 10x (bar = 100 μm) and 40x (bars = 50 μm) magnification. The statistically significant differences (*p* < 0.05) were represented by the ^*^ in comparison with the control group MPLA in which ^*^p < 0.05, ^**^p < 0.01, ^***^p < 0.001.

**Figure 7:**
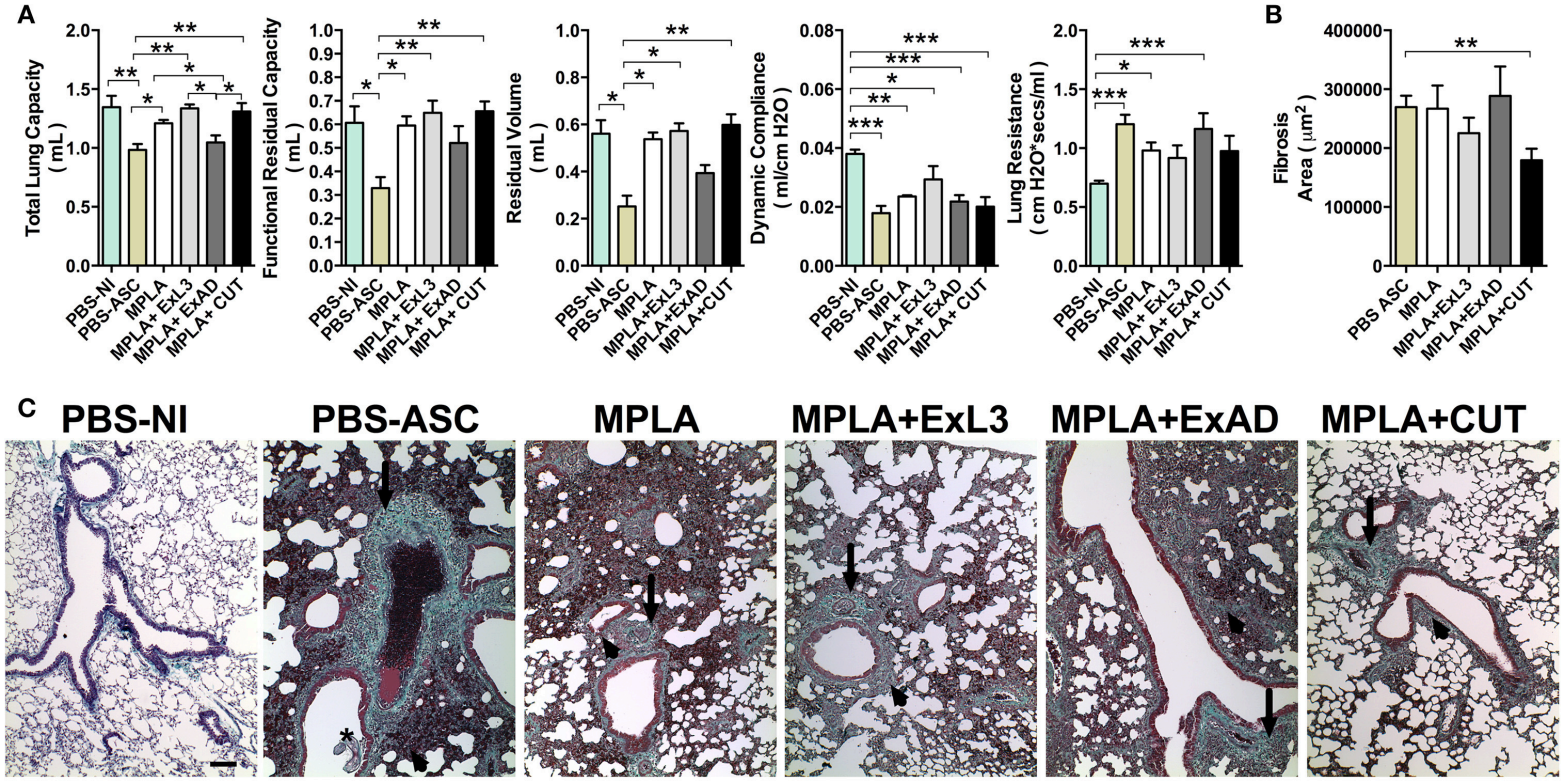
Immunization with different *A. suum* extracts had distinct effects on pulmonary fibrosis and dysfunction following *A. suum* infection. **(A)** Determination of lung dysfunction induced by *A. suum* infection by spirometry; **(B)** Morphometry of fibrosis area; and **(C)** Histopathological picture from lungs. Deposition of fibrous connective tissue around vessels (arrow), deposition of fibrous connective tissue around airways (arrowhead). Stained by Gomori's thrichrome, 10X (bar = 100 μm). The statistically significant difference was measured by the Kruskal-Wallis test and Dunn's multiple comparison test. ^*^p < 0.05, ^**^p < 0.01, ^***^p < 0.001.

Next, the lung tissue from mice immunized with the *A. suum* antigens and subsequently infected by the parasite eggs were stained with Hematoxylin & Eosin to further evaluate lung injury and inflammation (Figure 6) and with Gomori's trichrome for assessment of collagen deposition and fibrosis (Figure 7). The histopathological score revealed increased total pulmonary tissue inflammation and hemorrhage in all groups challenged with *Ascaris suum* compared to PBS-NI mice (Figure 6A). However the CUT-immunized mice showed reduced tissue damage compared to the PBS-ASC group, with less peribronchial, perivascular, and parenchymal inflammation (Figure 6A) and reduced area of inflammation by morphometry (Figure 6B). In the PBS-ASC group (Figure 6C), the presence of perivascular edema (arrow) with hemorrhagic areas and predominantly polymorphonuclear inflammatory infiltrate, characterized by eosinophils and neutrophils was observed in peribronchial areas (red arrowhead). We also observed larvae around the hemorrhagic areas caused by worm transmigration, and occupying the lumen of the bronchi (asterisk) and bronchioles. Hypertrophy and hyperplasia of bronchial epithelial (arrowhead) cells were frequently observed and leukocytes were present in the airways. The *Ascaris* infected MPLA adjuvant group demonstrated intense perivascular edema (arrow) and a mixed peribronchial inflammatory infiltrate composed of eosinophils, neutrophils, and macrophages (red arrowhead) as well as mucous plugging obstructing the bronchus (hashtag). Areas of multifocal hemorrhage, mainly due to the tissue damage induced by the migration of the larvae throught the organ, were also noted (Figure 6C). The histopathological analysis of the immunized groups showed differences in the tissue injury. The lungs of mice immunized with ExL3 and ExAD demonstrated significant increases in inflammatory infiltrates, which were composed predominantly of macrophages and eosinophils, with minimal neutrophils and lymphocytes in the peribronchial areas (Figure 6C) (red arrowhead). Moreover, the reduction in the alveolar space caused by tissue inflammation and hemorrhage was more prevalent in the groups immunized with ExL3 and ExAD. However, in comparison to ExAD, there was no perivascular edema evident (arrows). Additionally, hypertrophy and hyperplasia of bronchial epithelial cells (arrowhead) was found only in the ExAD group, but not in the ExL3 immunized mice. Finally, the CUT-immunized group (Figure 6C) demonstrated reduced mixed, peribronchial inflammatory infiltrate with predominance of lymphocytes and macrophages and minimal eosinophils and neutrophils (red arrowhead) and reduced perivascular edema (arrows). Additionally, the CUT-immunized group demonstrated increased areas of preserved lung architecture and reduced hypertrophy and hyperplasia of bronchial epithelial cells (arrowhead).

### Immunization with different *A. suum* extracts had distinct effects in pulmonary fibrosis and dysfunction following *A. suum* infection

As previously described, *A. suum* infection induces severe pulmonary dysfunction due to larval migration (20). The respiratory mechanics including Total Lung Capacity (TLC), Functional Residual Capacity, (FRC), Residual Volume (RV), elasticity by Dynamic Compliance (Cdyn), and Resistance of Lung (RI) were assessed at 8 days post-infection (Figure 7A). *A. suum* challenge led to reduction in pulmonary volumes, as depicted by loss of TLC, FRC, and RV compared to PBS-NI or MPLA adjuvant groups. Moreover, the analysis of TLC, FRC, and RV showed that ExL3- and CUT-immunized mice were protected from the loss of pulmonary volume induced by *A. suum* challenge, whereas the ExAD-immunized mice were not (Figure 7A). However, a significant reduction (*p* < 0.001) in Lung Compliance (Cdyn) was observed in all *Ascaris*-challenged groups, even after antigen immunization (Figure 7A). Lung Resistance from mice challenged with *A. suum* (PBS-ASC), MPLA-adjuvant and ExAD-immunized mice was significantly increased compared to PBS-NI (Figure 7A), but not the ExL3- and CUT-immunized mice.

*Ascaris* challenge additionally causes significant tissue damage and remodeling, leading to aberrant pulmonary scarring, as observated by Gomori's thrichrome stain (Figure 7B). The PBS-ASC, MPLA-immunized, and ExAD-immunized mice followed by *Ascaris* challenge displayed diffuse lung fibrosis, characterized by collagen deposition in perivascular (arrows) and peribronchial (arrowhead) spaces (Figure 7C), as result of worm transmigration into airways (asterisk). Conversely, the CUT-immunized mice showed a reduction in lung collagen (Figure 7B) with focal perivascular (arrows) and peribronchial (arrowhead) deposition (Figure 7C).

### Transfer of igg antibodies protects mice against *ascaris suum* infection

**Figure 8:**
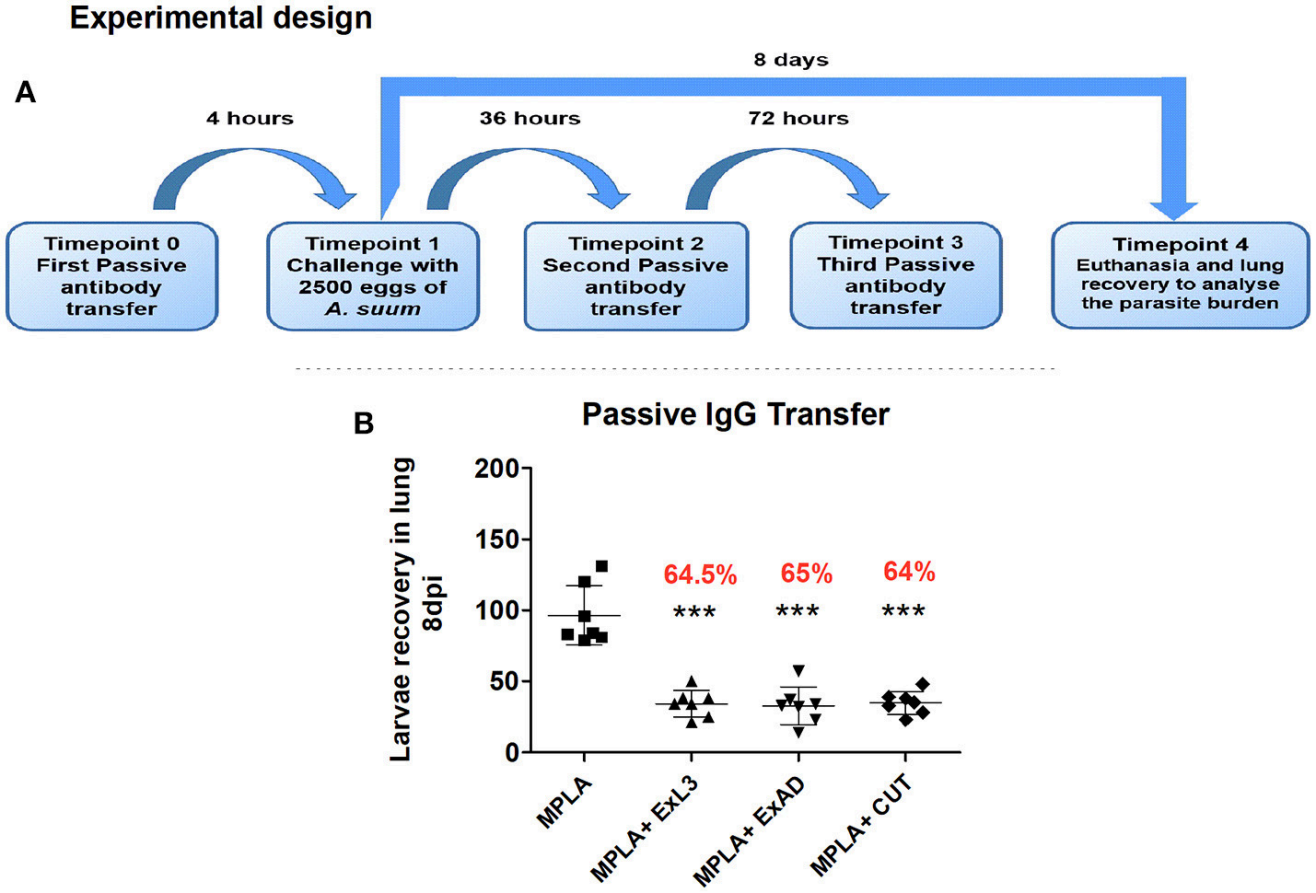
Passive transfer of IgG antibodies protects mice against *Ascaris suum* infection. Anti-*A. suum* IgG antibodies purified through protein A affinity columns were administered to BALB/c mice. **(A)** Experimental design for the passive transfer of anti-*Ascaris* IgG antibodies. **(B)** Number of larvae recovered in the lung tissue of mice after 8 days of infection. Statistically significant differences (*p* < 0.05) were plotted compared to the control group MPLA in which ^***^p < 0.001.

To test the hypothesis that the humoral immune response induced by antigen-specific immunization protects mice from subsequent *A. suum* infection, passive transfer of purified IgG antibodies from immunized mice to naïve and subsequently infected mice was carried out (Figure 8A). Mice that passively received anti-antigen IgG antibody showed a significant reduction in the number of migrating larvae in the lungs compared to the control group (Figure 8B). IgG antibodies from the group immunized with ExL3 provided protection of 64.5% (*p* < 0.05) while IgG from the groups immunized with ExAD and CUT provided 65% and 64% (*p* < 0.05) protection, respectively, compared to control antibodies from mice immunized with MPLA adjuvant.

## Discussion

The present work is the first to detail the immunologic and clinical impact of immunization with crude extracts from different *Ascaris* stages and structures including ExL3, ExAD, and CUT, leading to protection from subsequent *A. suum* infection in mice. The selected antigens were evaluated to determine their immunogenic capabilities against ascariasis, focusing on morbidity and infection control. This study found that immunization of mice with ExL3, ExAD, and CUT extracts induced increased systemic IL-5 levels, lung IL-5 levels, eosinophil, and macrophage influx into the airways, and antigen-specific IgA, IgG, and IgE production, leading to reduced parasite burdens, reduced pulmonary dysfunction and preservation of lung architecture in response to subsequent *A. suum* challenge. As a proof of concept, this study additionally demonstrated that administration of antigen-specific IgG, purified from mice immunized with ExL3, ExAD, or CUT, to naïve mice provided protection from subsequent *Ascaris* challenge. Moreover, CUT-immunized mice showed increased IL-10 levels in blood and lung tissue, as well as reduced polymorphonuclear leukocytes and increased lymphocytes in the airways. Additionally, CUT immunization was associated with reduced lung function, including attenuated pulmonary inflammation and fibrosis after subsequent *Ascaris* challenge, suggesting CUT vaccine may not only induce immunologic protection but also prevents significant pulmonary injury when compared to other antigen vaccines.

Previous studies have shown that purified proteins from *A. suum* extracts may induce immune protection in the host (15–17), suggesting potential immunogenic components of the parasite capable of inducing this protective responses against *Ascaris* infection. Enolase enzyme, the gene encoding *Ascaris suum* enolase (As-enol-1), has been considered a potential vaccine candidate against ascariasis because it is capable of eliciting a Th1/Th2 immune response, affording host protective immunity against parasite larval migration (17). Additionally, immunization with a 14-kDa surface protein of *A. suum* (As14) combined with Cholera B toxin causes significant increase in IgE and IgG levels in the serum and mucosa, and was associated with a 64% reduction in larvae burden in the lungs compared to non-vaccinated groups (16). Other proteins derived from parasite extracts, such as As16, As37, and As24, are also being tested as possible vaccine candidates (8, 14). Specifically, immunization with As16 formulated with Montanide ISA720 adjuvant induces a Th2-skewed immune response, stunted larval development and significant larvae reduction (36.7%) in the lungs of immunized mice after *A. suum* challenge (15).

In the present study, three different *Ascaris* extract vaccines demonstrated significant induction of humoral responses, with marked systemic production of IgE and IgGs, including specificity to As14 and As16 antigens, as well as increased airway IgA using MPLA as an adjuvant after three different *Ascaris* vaccines. In fact, *Ascaris* extracts adjuvanted with MPLA lead to subsequent protection against *Ascaris* challenge, with marked reductions of larval burdens in the lungs of 61.4% for ExL3, 59.1% for CUT, and 51.4% for ExAD. The reduction in larval burdens in the lungs in vaccinated mice after *Ascaris* challenge corroborates with other studies that used parasite-derived proteins for vaccination, in which reductions of 64.13 and 88.62% were obtained (7, 8). However, despite the large reduction in parasite burden using parasite-derived proteins for immunization, the mechanism of immunologic and clinical efficacy remains unknown.

To investigate the possible immunologic mechanism we confirmed the concentration of extract-specific IgG levels for all vaccine formulations. We then investigated if passive transfer of IgG from immunized animals into naïve animals would provide similar protection against subsequent *Ascaris* challenge. We transferred purified IgG antibodies, obtained from the serum of immunized mice, into WT BALB/c naive mice, and evaluated the parasite burdens on the eighth day post-infection. Interestingly, a significant reduction (65%) in parasite burdens was observed in all immunized groups, suggesting a crucial and important role of the humoral response in reducing larval migration and subsequent larval burden in the lungs. Similar evidence supporting the importance of humoral immunity for parasite control was documented in a *Strongyloides ratti* model, in which passive transfer of *Strongyloides ratti* specific-IgM led a significant reduction in larval migration and adult *S. ratti* development (22). Likewise, IgG antibodies from mice immunized with irradiated *Schistosoma* cercariae were used for passive transfer in a *Schistosoma mansoni* infection model, which again demonstrated a reduction of 20–50% in cercariae after infection when compared to the control group (23). While the protective role of antibodies is controversial during natural *Ascaris* infection (24, 25), our results demonstrate an unequivocal protective effect of antibodies derived from vaccination with crude extracts, indicating that specific humoral responses induced by vaccination may form an important arm of immunity against *Ascaris* infection.

After immuninzation with all crude extracts, there was a significant increase in inflammatory infiltrates following *Ascaris* challenge, as well as increased thickening of the interalveolar septa, reduced free alveolar space, and increased lung fibrosis. These findings are similar to those of a *Toxocara* study that demonstrated increased intense local inflammatory infiltrates after infection with the parasite due to the larvae migrating through the organ (26). Moreover, the alveolar septa thickening and fibrosis, alveolar edema, hemorrhage, and airways exsudation are the most common histopathological manifestations in the lungs during infection with the larval stage of *A. suum*, resulting in pulmonary dysfunction. Immunization of mice with specific antigenic extracts, followed by *A. suum* challenge, resulted in a varying degree of inflammatory infiltrates and lung function depending on the extract. We found that immunization with either ExL3 or CUT resulted in reduced granulocyte influx into the airways and improved lung function compared to healthy controls. The preservation of lung physiology in ExL3 and CUT immunized mice likely reflects the significant reduction in parasite burdens allowing for suppression of lung damage and maintenance of lung tissue integrity.

The profile of serum cytokines was characterized by the quantification of IL-2, IL-5, IL-6, IL-13, TNF-α, and IL-10, in immunized mice post-*Ascaris* challenge. Acute infection by *Ascaris* and other helminths generally induces mixed production of Th1/Th2 or Th2/Th17 cytokines, with significant elevation of IL-5, IL-10, and IFN-γ in blood (20, 27–29). During the progress of the lifecycle, helminths may regulate the host immune response through induction of the secretion of regulatory cytokines, such as IL-10 and TGF-β, mostly derived from macrophages and Tregs (30). CUT-immunized mice displayed increased IL-10 levels, suggesting that *A. suum* vaccination can stimulate regulatory cytokines. In addition, helminth co-infections are characterized by high IL-10 and IL-5 (31), suggesting an immunomodulatory role of helminth infections. Similarly, our study demonstrates an increased production of IL-5 and IL-10 in serum and in lung tissue after immunization and subsequent *Ascaris* challenge. Interestingly, IL-5, a Th2 cytokine, known to be a potent inducer of B cell differentiation, antibody secretion, and isotype switching (32), and has been previously described in experimental models of *A. suum* infection (20). Therefore, a Th2 protective response triggered by immunization may be secondary to an IL-5 driven, IgG specific humoral response. This hypothesis was tested by evaluating the impact of passive transfer of anti-antigen IgG antibodies to naïve mice, followed by *Ascaris* challenge. Transference of anti-antigen IgG was sufficient to attenuate the immunologic response induced by *Ascaris* challenge, suggesting that the observed reduction in parasite burdens was mediated by the humoral immune response. Further studies are underway that evaluate the interplay between type 2 cytokines and the humoral immune response, in order to determine the mechanisms of protection.

Finally, we evaluated the reactivity of antibodies from ExL3-, ExAD-, and CUT-immunized mice to two promising *A. suum* recombinant protein vaccine candidates. The results revealed strong recognition of As16 by antibodies from ExAD- and CUT-immunized mice compared to ExL3-immunized mice and controls and only weak responses to As14. Interestingly, when As14 and As16 were tested in vaccine efficacy trials (15), immunization with As16 induced a significant reduction in the parasite burden after challenge, while immunization with As14 provided only minimal protection. Recognition of As16, an important antigen for *Ascaris* subunit vaccine development, in the crude extract demonstrates the efficacy of this vaccine design and antigens selection.

In this study, we demonstrated that vaccination against *Ascaris* infection induced protective IgG antibodies that reduced the parasite burdens of infected mice. The CUT vaccine is of particular interest for the development of future vaccines, as it induced IL-5 and an extract-specific humoral response that resulted in reduced parasite burdens, as well as reduced pulmonary inflammation, fibrosis, and dysfunction. Further studies are underway to investigate the IL-10-related immunoregulatory mechanisms induced by the CUT vaccine. We suggest that this crude extract immunization model is a useful tool for screening and identifying novel *Ascaris* antigen vaccine candidates to determine their antigenic potential and immunogenic relevance. Using this animal model, we aim to identify an *Ascaris* vaccine target that could be formulated with other helminthic antigens, in order to create a panhelminthic vaccine for humans or animals that could significantly reduce global morbidity in *Ascaris*-endemic regions.

## Author contributions

Conceived and designed the experiments: AG-G, PG-G, RR, LB, and RF. Performed the experiments: AG-G, DN, PG-G, FB, RR, FO, LK, MSC, CA, and MVC. Analyzed the data: AG-G, DN, PG-G, LB, MVC, RR, RF, KJ, JW, MB, PH, BZ, and DB. Contributed reagents, materials, analysis tools: AG-G, PG-G, RR, LB, RF, DB, MA, and PH. Wrote and reviewed the paper: AG-G, PG-G, RR, LB, RF, FO, KJ, JW, MB, PH, and BZ.

### Conflict of interest statement

The authors declare that the research was conducted in the absence of any commercial or financial relationships that could be construed as a potential conflict of interest.

## Supplementary material

Supplementary Figure 1 Experimental groups and experimental design of *Ascaris* immunization. Experimental design of the protocol of active immunization of the animals with the crude antigens of larvae, crude extract of adult worm and crude extract of cuticle of *A. suum* and subsequent infection with infective eggs of the parasite.

Supplementary Figure 2 Electrophoretic profile of *A. suum* antigens. SDS polyacrylamide gel electrophoresis bands profile (SDS-PAGE) of the crude extract of infective larvae (ExL3), crude extract of adult worm (ExAD) and crude extract of adult worm cuticle (CUT).

Supplementary Table 1 Histopathological scoring system for mouse lungs.
