# Supplementary figures and images for: IgG Induced by Vaccination With Ascaris suum Extracts Is Protective Against Infection
Source: Front Immunol. 2018 Nov 9;9:2535. doi: 10.3389/fimmu.2018.02535 (PMC6238660; doi:10.3389/fimmu.2018.02535)

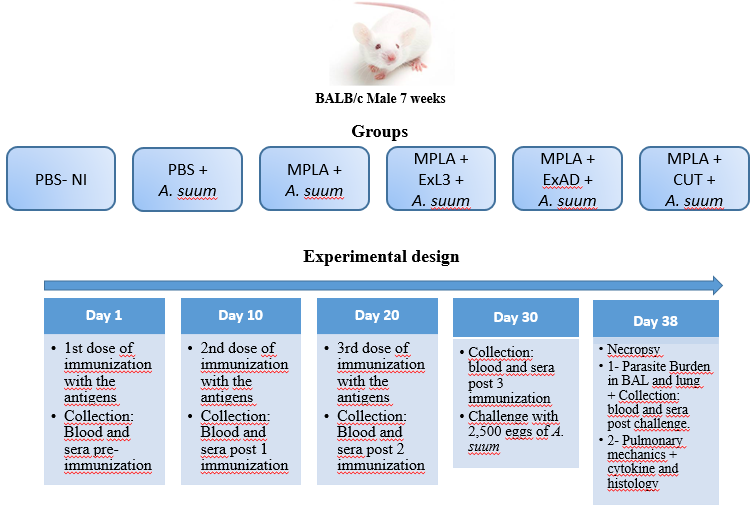

Supplement: Supplementary Figure 1 — Experimental groups and experimental design of Ascaris immunization. Experimental design of the protocol of active immunization of the animals with the crude antigens of larvae, crude extract of adult worm and crude extract of cuticle of A. suum and subsequent infection with infective eggs of the parasite. [file Image_1.TIF]

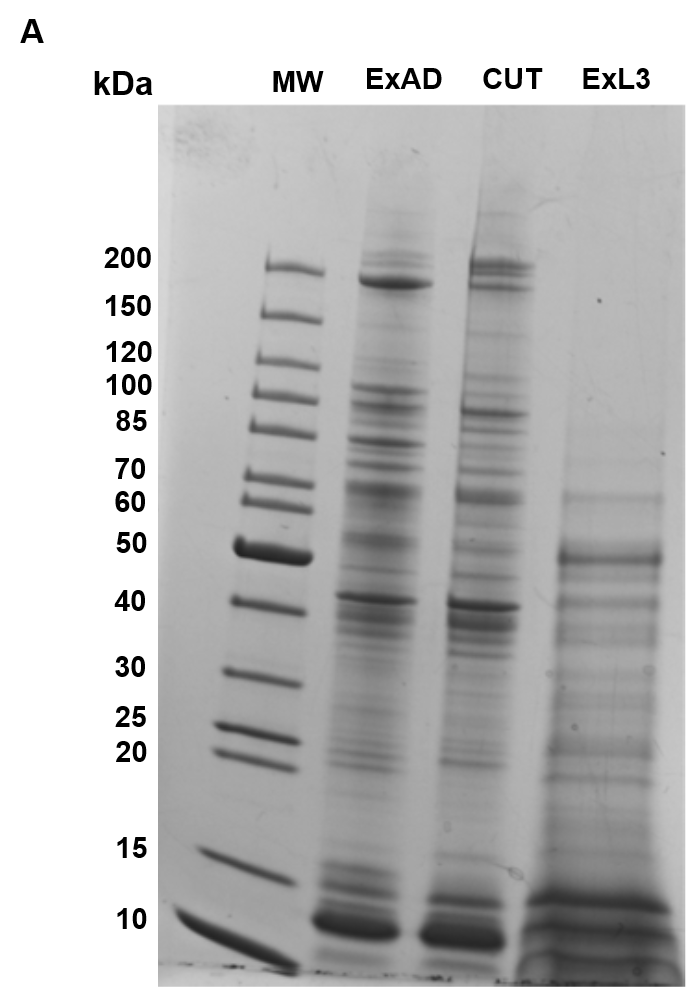

Supplement: Supplementary Figure 2 — Electrophoretic profile of A. suum antigens. SDS polyacrylamide gel electrophoresis bands profile (SDS-PAGE) of the crude extract of infective larvae (ExL3), crude extract of adult worm (ExAD) and crude extract of adult worm cuticle (CUT). [file Image_2.TIF]
