# Supplementary material for: IgG Induced by Vaccination With Ascaris suum Extracts Is Protective Against Infection
Source: Front Immunol. 2018 Nov 9;9:2535. doi: 10.3389/fimmu.2018.02535 (PMC6238660; doi:10.3389/fimmu.2018.02535)
Supplement: Supplementary Table 1 — Histopathological scoring system for mouse lungs. [file Data_Sheet_1.PDF]

Supplemental Table 1. Histopathological Scoring System for Mouse Lungs

**Score 1)**	
**Airways Inflammation**	**Score /5**
0 = Lack of inflammatory cells around airways - Absent	
1 = Some airways have small numbers of cells - Mild	
2 = Some airways have significant inflammation. - Moderate	
3 = Majority of airways have some inflammation. - Marked	
4 = Majority of airways are significantly inflammed - Severe	
5 = All of airways are completely inflammed. – Whole	
**Score 2)**	
**Vascular Inflammation**	**Score /5**
0 = Lack of inflammatory cells around vessels. - Absent	
1 = Some vessels have small numbers of cells. - Mild	
2 = Some vessels have significant inflammation. - Moderate	
3 = Majority of vessels have some inflammation. - Marked	
4 = Majority of vessels are significantly inflammed - Severe	
5 = All of vessels are completely inflammed. - Whole	
**Score 3)**	
**Parenchymal Inflammation (at 10X magnification)**	**Score /5**
0 = <1% affected	
1 = 1-9% affected	
2 = 10-29% affected	
3 = 30-49% affected	
4 = 50-69% affected	
5 = >70% affected	
